# Diagnostic Yield of Echocardiography in Syncope Patients with Normal ECG

**DOI:** 10.1155/2016/1251637

**Published:** 2016-01-04

**Authors:** Nai-Lun Chang, Priyank Shah, Sharad Bajaj, Hartaj Virk, Mahesh Bikkina, Fayez Shamoon

**Affiliations:** ^1^Department of Internal Medicine, St. Joseph's Regional Medical Center, Paterson, NJ 07503, USA; ^2^Department of Cardiology, St. Joseph's Regional Medical Center, Paterson, NJ 07503, USA

## Abstract

*Aim*. This study aimed to assess the role of echocardiography as a diagnostic tool in evaluating syncope patients with normal versus abnormal electrocardiogram.* Methods*. We conducted a retrospective study of 468 patients who were admitted with syncope in 2011 at St. Joseph's Regional Medical Center, Paterson, NJ. Hospital records and patient charts, including initial emergency room history and physical, were carefully reviewed. Patients were separated into normal versus abnormal electrocardiogram groups and then further divided as normal versus abnormal echocardiogram groups. Causes of syncope were extrapolated after reviewing all test results and records of consultations.* Results*. Three hundred twelve of the total patients (68.6%) had normal ECG. Two-thirds of those patients had echocardiograms; 11 patients (5.7%) had abnormal echo results. Of the aforementioned patients, three patients had previous documented history of severe aortic stenosis on prior echocardiograms. The remaining eight had abnormal but nondiagnostic echocardiographic findings. Echocardiography was done in 93 of 147 patients with abnormal ECG (63.2%). Echo was abnormal in 27 patients (29%), and the findings were diagnostic in 6.5% patients.* Conclusions*. This study demonstrates that echocardiogram was not helpful in establishing a diagnosis of syncope in patients with normal ECG and normal physical examination.

## 1. Introduction

Syncope, a common clinical syndrome, is characterized by transient loss of consciousness and postural tone caused by brief global cerebral hypoperfusion from decreased cardiac output and/or peripheral resistance. It is of rapid onset, brief duration, and spontaneous and complete recovery. Syncope accounts for about 1% of all hospital admissions and 3% emergency department visits and annually costs approximately $2 billion in the United States [1–4].

Causes of syncope are vast and often present as a diagnostic challenge for physicians, resulting in extensive workup. The causes could be vascular, cardiac, neurologic, metabolic, psychologic, and finally of unknown origin. As per Linzer et al., the most common causes for syncope are unknown origin (34%), vasovagal and orthostatic hypotension (26%), and arrhythmias (14%) [5]. Several guidelines exist to assist physicians approach the evaluation of syncope in an algorithmic way [6–8]. Echocardiography (Echo) is a common imaging modality used in the workup of patients presenting with syncope. According to American College of Cardiology 2011 Appropriate Use Criteria for Echocardiography, echo is not necessary in patients with lightheadedness and/or presyncope who do not have history of cardiac disease. However, in syncope patients who have no previous heart disease, the use of echocardiography as one of the diagnostic tools is still appropriate [9]. In 1995, Recchia and Barzilai conducted a retrospective review at an urban university hospital. They considered the following ECG changes as significant: Q waves suggesting prior myocardial infarcts, bundle branch block, ventricular ectopy or arrhythmia, and Mobitz II or higher degrees of atrioventricular nodal block. From chart review of 128 syncope patients, more than 60% of the patients underwent echocardiography and more than half the results were normal. No echo reports showed unsuspected causes of syncope. They concluded that echocardiogram did not add to any additional diagnostic value for patients without suspected cardiac disease [10]. Another retrospective study of 323 patients by Anderson et al. in 2012 suggested that echo may be a poor utilization of resources for patients with normal ECG due to very low yield of structural heart disease in such patients. Eighty-eight percent of the 267 patients with normal ECG had echocardiogram done to evaluate cardiac structure. However, none showed structural abnormality [11].

Echocardiography seems to have over time become a routine test ordered to assess a wide array of cardiac diseases. Several studies have shown that echocardiogram has been performed in more than half of the patients with complaints of syncope [10, 12]. We suggest that the most important diagnostic tool in evaluation of syncope is a detailed history and physical exam. The primary objective of our study was to determine the diagnostic yield of echo in patients presenting with syncope, particularly in those with normal electrocardiogram and physical exam not suggestive of cardiac disease.

## 2. Methods

This is a retrospective, observational study at St. Joseph's Regional Medical Center, a quaternary academic care and state-designated trauma center in Paterson, New Jersey. Data were collected through a retrospective chart review of all adult patients admitted for syncope during a 12-month period from January 2011 to December 2011. Cases were identified based on the initial diagnosis made by physician on admission. We collected and reviewed the following data from medical charts and electronic medical records: history of presenting illness and associated symptoms, demographic data, medical history, and results of ECG and echocardiogram.

The initial ECG was performed on admission. We defined abnormal ECG and echo as the following (Table 1). ECGs showing arrhythmias, Q waves, ischemic changes, 2nd- and 3rd-degree AV block, paced rhythm, QTc > 500 ms, left bundle branch block, bifascicular block, Brugada pattern, and abnormal axis were considered abnormal. All echocardiograms were performed by registered diagnostic sonographers. Echocardiograms demonstrating ejection fraction less than 45%, any severe valvular abnormalities, severe ventricular wall hypertrophy, septal wall motion abnormality, hypertrophic cardiomyopathy with outflow tract obstruction, severe pulmonary hypertension, and hemodynamically significant pericardial effusions were considered abnormal.

Both ECG and echo reports were read and approved by an attending cardiologist. The reports were then reviewed by investigators.

Initial history and physical exam in the emergency room and the results of all diagnostic tests and records of all consultations were reviewed. The final cause of syncope was assigned after careful review of all physicians' notes and test results. We looked to see the number of patients admitted with syncope who had ECG and echocardiogram and the number of patients who had abnormal ECG and/or abnormal echo.

## 3. Results

**Figure 1:**
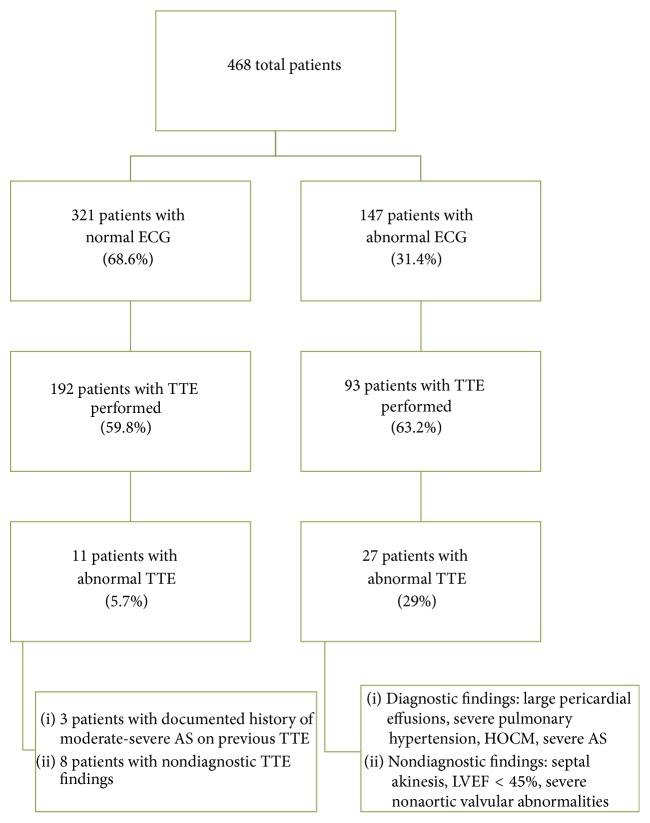
Patient segregation flowchart. ECG, electrocardiogram. TTE, transthoracic echocardiogram. HOCM, hypertrophic cardiomyopathy. AS, aortic stenosis.

Out of the 468 patients, we had 321 patients (68.6%) with normal ECG. One hundred ninety-two echocardiograms (59.8%) were done in patients with normal ECG reading (Figure 1). Of those, 11 patients (5.7%) had abnormal TTE. However, 3 patients had prior documented echo results and history and physical findings of severe aortic stenosis. Discharge diagnoses were orthostatic hypotension and unknown causes. In comparison, in the group of 147 patients (31.4%) with abnormal ECG, 93 echocardiograms (63.2%) were done, and 27 of them (29%) were abnormal. However, the finding of structural abnormalities on echo does not establish the cause for the patient's syncopal episode.

Echocardiogram by itself did not help in determining the cause of syncope in any patient in normal ECG group. Three of the eleven patients with abnormal echocardiograms had prior documentation of severe aortic stenosis. The other eight patients, which are 4% of the patients with normal ECG with abnormal TTE, had abnormal echo findings showing reduced LVEF less than 45% or other nonaortic valvular pathology. Two patients had vasovagal response; one patient had orthostatic hypotension. Five patients had unknown cause upon chart review (Table 2). However, echo did not help in establishing diagnosis in any of those patients.

Of the abnormal echocardiograms in the abnormal ECG group, besides the nondiagnostic findings, there were one patient with severe pulmonary hypertension, two patients with significant pericardial effusions, one patient with severe aortic stenosis, one patient with significant septal motion abnormality, and one with hypertrophic cardiomyopathy. Those are all diagnostic causes of syncope (Table 2). Hence, in the abnormal ECG group, echo aided in the diagnosis in 6.5% patients (6 out of 93).

In patients with TTE performed, nonspecific causes, vasovagal response, and orthostatic hypotension all together comprise 91.6% and 68.8% of the discharge diagnosis in normal ECG and abnormal ECG groups, respectively (Table 3).

In the normal ECG group, the six patients found to have arrhythmia (i.e., paroxysmal atrial fibrillation) or conduction disease (i.e., sinus node dysfunction and severe His-Purkinje conduction disease) as possible causes of their syncopal episode, echocardiogram did not show significant cardiac structural abnormalities.

## 4. Discussion

In clinical practice, echocardiography has become one of the most routinely ordered modalities for assessing the cause of presyncope and syncope. Recchia and Barzilai [10] found that echocardiography was performed in more than half of the patients with complaints of syncope but no finding had any diagnostic value. Several studies have shown that younger patients, patients with no significant cardiac history, have low echocardiographic yield. Panther et al. [13] demonstrated in a 7-year retrospective study of 439 patients, who were referred for echocardiographic studies in evaluation of syncope, patients younger than 40 years of age with syncope will most likely have normal echocardiography. In age groups 40–59 and 60+, 58.2% and 29.6% had normal echocardiographic findings. In a prospective, observational study done by Sarasin et al. [14] in 2002, 155 of 650 patients (23%) presented with syncope without clear cause. Routine echocardiography showed no diagnostic cause to explain the event. In patients without significant cardiac history or abnormal ECG, echocardiography was nondiagnostic or normal. Thirteen percent of the patients had positive cardiac history or abnormal ECG but echocardiogram only showed left ventricular systolic dysfunction or minor nonrelevant findings. They concluded that echocardiography was most useful when its use was restricted to high-risk patients with significant history of cardiac disease and ECG abnormalities.

Results of our study prove that echocardiogram has a low overall diagnostic yield in syncope patients without a history of cardiac structural disease. Eleven patients with normal ECG (5.7%) had abnormal TTE. Three of those already had previous documentation of severe aortic stenosis on prior history and physical exam and echocardiograms. Remaining eight patients' echocardiogram did not uncover any intracardiac abnormality to have caused the syncopal episode. Hence the diagnostic value of echo in patients with normal ECG and normal physical exam was 0%. For patients with abnormal ECG, echo aided in diagnosis in 6.5% patients. Majority of the patients from either group had discharge diagnosis as syncope due to nonspecific or undetermined causes.

In agreement with other similar studies, we reason that echocardiography should not be routinely used in patients presenting with syncope particularly in those with normal ECG and physical exam not suggestive of cardiac disease. Echocardiogram on every patient that comes with complaints of syncope is not cost effective.

## 5. Limitations

For one, this is a retrospective study. We do not know the outcomes of the patients after their discharge. Second, 129 of the 321 patients (40%) from the normal ECG group did not have an echocardiogram done on that admission. If echocardiogram was done, the percentage of abnormal finding may be higher. Third, we depended on emergency room records for the initial history and physical examination. Details could have been missed. Reviewing of the charts further strengthens the call for more detailed history and physical exam upon initial contact of patients.

## 6. Conclusions

Echocardiography should not be a routine diagnostic tool used to elucidate causes of syncope unless causes were unexplained by history and physical exam and patients have significant cardiac history with abnormal ECG.

## Figures and Tables

**Table 1:** Definitions of abnormal ECG and echo.

Abnormal ECG	Abnormal echo
(i) Arrhythmias (ii) Q waves (iii) Ischemic changes (iv) 2nd- and 3rd-degree AV block (v) Paced rhythm (vi) QTc > 500 ms (vii) Left bundle branch block (viii) Bifascicular block (ix) Brugada pattern (x) Abnormal axis	(i) LVEF < 45% (ii) Moderate-severe valvular abnormalities (stenosis or regurgitation) (iii) Severe ventricular wall hypertrophy (iv) Any septal wall motion abnormality (v) Hypertrophic cardiomyopathy with outflow tract obstruction (vi) Severe pulmonary hypertension (vii) Hemodynamically significant pericardial effusions

AV, atrioventricular. LVEF, left ventricular ejection fraction.

**Table 2:** Echocardiographic findings in patients with abnormal TTE in both normal and abnormal ECG groups.

	TTE findings	Discharge diagnosis	Echo useful in diagnosis of syncope?
Normal ECG with abnormal TTE (*n* = 11)	Moderate-severe aortic stenosis with mild aortic insufficiency	Orthostatic hypotension	No^*∗*^
	Severe aortic stenosis	Multifactorial nonspecific cause	No^*∗*^
	Severe aortic stenosis. Normal LV function with EF 55–60%. Mild concentric LVH.	Undetermined cause	No^*∗*^
	Right atrial enlargement. Right ventricular hypertrophy. Severe tricuspid regurgitation. Mild mitral regurgitation.	Orthostatic hypotension	No
	LVEF 35–40% with grade I diastolic dysfunction	Unknown	No
	Estimated left ventricular ejection fraction of 40%. Mild mitral regurgitation.	Unknown	No
	LVEF 40% diffuse hypokinesis	Unknown	No
	LV cardiomyopathy, LVEF 35–40%	Unknown	No
	Moderate global hypokinesis of the left ventricle with ejection fraction 35%. Tricuspid regurgitation	Unknown	No
	Moderate LV dysfunction with EF 40% and diffuse hypokinesis. Aortic root calcification.	Vasovagal response	No
	Mild dilated LV with reduced systolic function. EF 35%. Moderate global hypokinesis.	Vasovagal response	No

Abnormal ECG with abnormal TTE (*n* = 27)	Mild mitral valve prolapse. Concentric LVH. Septal hypokinesis. Moderate left atrial enlargement. Severe pulmonary hypertension.	Severe pulmonary hypertension	Yes
	Severe MR, severe TR	Orthostatic hypotension	No
	Patient in atrial fibrillation rhythm. Moderate hypokinesis of the LVEF 35–40%.	Unknown	No
	LVEF 40%	Atrial flutter with RVR	No
	Mildly dilated left ventricle with severely reduced systolic function and LVEF of 25–30%. Moderate concentric left ventricular hypertrophy.	Unknown	No
	LVEF of 40–45%. Akinesis of the anterior septum and inferior septum. Trace MR and TR.	Vasovagal response	No
	LV enlargement. Generalized hypokinesis. EF ~25%.	Unknown	No
	LVEF 30%. Diffuse hypokinesis	Acute HF exacerbation with respiratory failure	No
	Moderate AI and TR. LVEF 30%, LVH	Carotid hypersensitivity	No
	LV enlargement with generalized hypokinesis and EF 25–30%	Unknown	No
	Moderate LA enlargement. Moderate-severe MR.	Unknown	No
	Dilated LV with EF ~25%	Unknown	No
	Marked anteroapical hypokinesis with LVEF ~30%. Mild-Moderate AI.	Unknown	No
	LVH. Normal EF. Large significant pericardial effusion.	Large pericardial effusion	Yes
	LVEF 30%. LVH.	Vasovagal response	No
	LVEF 30% with diffuse hypokinesis.	Vasovagal response	No
	LV enlargement with generalized hypokinesis. EF 25–30%	Ventricular fibrillation status after ICD shock	No
	Generalized hypokinesis. Paradoxical septal motion. LVEF 25%, MR and TR	Paradoxical septal motion	Yes
	Left ventricular hypertrophy. Paradoxical motion of interventricular septum with preserved LV systolic function. Mild to moderate tricuspid insufficiency	Sinus bradycardia secondary to medication overdose	No
	LVEF severely reduced and estimated at 25%. Severe global hypokinesis of the left ventricle. The entire inferior wall is akinetic. Impaired left ventricular relaxation. Severe mitral regurgitation.	Unknown	No
	Severe aortic stenosis. LVEF normal.	Severe aortic stenosis	Yes
	Asymmetrical septal hypertrophy of the left ventricle. Associated moderate left ventricular outflow tract gradient of 30 mmHg. Associated “pseudo-SAM” with no true systolic anterior motion of the mitral valve noted. Grade II left ventricular diastolic dysfunction.	HOCM	Yes
	Severe left ventricular dysfunction. Moderately severe to severe mitral regurgitation.	Severe conduction disease	No
	LVEF of 30%. Mild MR. Mild to moderate TR. Anteroapical hypokinesis and septal akinesis.	Acute HF exacerbation with respiratory insufficiency	No
	Dilated LV with EF 35%. Moderate global hypokinesis	Vasovagal response	No
	LVEF 35%. Inferior hypokinesis	Unknown	No
	LVEF of 35–45%. Findings compatible with pericardial effusion with right atrial cyclic compression.	Significant pericardial effusion	Yes

LVEF, left ventricular ejection fraction. AI, aortic insufficiency. MR, mitral regurgitation. TR, tricuspid regurgitation. LVH, left ventricular hypertrophy.

^*∗*^Previously documented AS on prior echo and hospital records.

**Table 3:** Causes of syncope in normal and abnormal ECG groups with TTE performed.

Cause of syncope	Normal ECG with TTE performed (%)	Abnormal ECG with TTE performed (%)
Vasovagal/orthostatic	58 (30.2)	32 (34.4)
Arrhythmia/conduction diseases	6 (3.13)	16 (17.2)
Symptomatic anemia	1 (0.5)	0
TIA/CVA	3 (1.5)	0
Any severe valvular diseases	3 (1.5)	1 (1.08)
Ischemic cardiac events/acute HF exacerbation	3 (1.5)	10 (10.8)
Device malfunction	0	1 (1.08)
Nonspecific	118 (61.5)	32 (34.4)
Carotid hypersensitivity	0	1 (1.08)
Total	192	93

TIA, transient ischemia attack. CVA, cerebrovascular accidents. HF, heart failure.
